# How Well Do U.S. Hispanics Adhere to the Dietary Guidelines for Americans? Results from the Hispanic Community Health Study/Study of Latinos

**DOI:** 10.1089/heq.2018.0105

**Published:** 2019-07-11

**Authors:** Anna Maria Siega-Riz, Nelson D. Pace, Nicole M. Butera, Linda Van Horn, Martha L. Daviglus, Lisa Harnack, Yasmin Mossavar-Rahmani, Cheryl L. Rock, Rocio I. Pereira, Daniela Sotres-Alvarez

**Affiliations:** ^1^ School of Nursing, University of Virginia, Charlottesville, Virginia.; ^2^ Exponent, Inc., Center for Health Sciences, Oakland, California.; ^3^ Department of Biostatistics, Collaborative Studies Coordinator Center, Gillings School of Global Public Health, University of North Carolina at Chapel Hill, Chapel Hill, North Carolina.; ^4^ Department of Preventive Medicine, Feinberg School of Medicine, Northwestern University, Chicago, Illinois.; ^5^ Institute for Minority Health Research, College of Medicine, University of Illinois, Chicago, Illinois.; ^6^ Division of Epidemiology and Community Health, School of Public Health, University of Minnesota, Minneapolis, Minnesota.; ^7^ Department of Epidemiology and Population Health, Albert Einstein College of Medicine, Bronx, New York.; ^8^ Department of Family Medicine and Public Health, University of California, San Diego, California.; ^9^ Department of Medicine-Endocrinology, Denver Health, Denver, Colorado.; ^10^ Division of Endocrinology, Metabolism and Diabetes, University of Colorado School of Medicine, Aurora, Colorado.

**Keywords:** diet, Hispanics, Dietary Guidelines, healthy eating

## Abstract

**Purpose:** To examine alignment between 2010 Dietary Guidelines for Americans (DGA) and dietary choices of individuals in the Hispanic Community Health Study/Study of Latinos (HCHS/SOL) between 2008 and 2011.

**Methods:** Data are from 15,633 adults 18–74 years from the population-based cohort in HCHS/SOL. The Healthy Eating Index (HEI) 2010 was used to measure diet quality. Means and standard errors (SEs) for the HEI total and each of the 12 component scores were calculated overall and by Hispanic/Latino heritage, sex, age group, and measures of acculturation. Linear regression was used to examine correlates of the HEI 2010 total score. All analyses accounted for complex survey design.

**Results:** The overall HEI mean of 63.8 (SE: 0.4) varied across groups from a high (healthier diet) of 71 (SE: 0.9) among Mexicans to a low of 56 (SE: 0.1) among Puerto Ricans. The proportion with a maximum score for the HEI components varied across heritage groups; >25% of adults adhered to recommendations for total proteins, and seafood and plant proteins, whole fruits, and greens and beans, with the exception of Cubans and Puerto Ricans, who had lower adherence scores for the latter two. The components with the lowest adherence were sodium (<2%) and fatty acids (overall 7.4%) among all heritage groups. Characteristics associated with better adherence included sociodemographic variables, Spanish language preference, weight status, medical conditions, and lifestyle behaviors.

**Conclusions:** Individuals with Mexican, Dominican, and Central American heritage had better overall dietary quality compared to other groups. However, all can improve their eating habits to align more with the DGAs by reducing sodium consumption and improving fatty acid ratios.

## Introduction

Dietary Guidelines for Americans (DGA) are revised on the basis of new evidence and published every 5 years by the federal government to promote healthy eating and physical activity levels for individuals 2 years of age and older.^1^ The recommendations are intended to accommodate the dietary preferences and traditions of various cultural, income, gender, and age subpopulations, but the current literature only minimally reflects current adherence in the Hispanic/Latino population. Data for all Americans (2009–10 National Health and Nutrition Evaluation Study [NHANES]) indicate less than ideal adherence to the 2010 Guidelines using the Healthy Eating Index (HEI) with scores ranging from ∼50 to 65 (out of 100) across all age groups, regardless of ethnicity.^2,3^ A recent study demonstrates that 7–10% of cardiometabolic deaths were associated with suboptimal intake of fruits, vegetables, seafood intake of omega-3 fats, and nuts/seeds, and a similar percentage from high intakes of sodium, processed meats, and sugar-sweetened beverages.^4^ More concerning is that all dietary factors, when considered together as a dietary pattern, were associated with ∼45% of all cardiometabolic deaths.^4^

The Hispanic Community Health Study/Study of Latinos (HCHS/SOL), the largest community-based cohort of Hispanic/Latino adults in the United States to date, assesses dietary intake using methods similar to those of NHANES (includes only Mexican Americans as the main group with the remainder of Hispanics lumped together as “other”). This provides a unique opportunity to evaluate Dietary Guidelines adherence among Hispanics/Latinos to fill in the gaps identified in the Scientific report of the 2015–2020 Dietary Guidelines Committee for this largest minority population and to identify diet components that might benefit from culturally tailored interventions (e.g., health promotion messages).^3^ Data are presented for major heritage groups (Cuban, Dominican, Mexican, Puerto Rican, Central American, and South American) and by sex and age. We also assess factors associated with adherence to understand the independent contributions of each factor and especially that of Hispanic/Latino heritage.

## Methods

### Sample population

The HCHS/SOL is a cohort study designed to identify disease prevalence rates and risk factors among Hispanic/Latino populations residing within four urban U.S. communities (Bronx, Chicago, Miami, and San Diego) and representing individuals with origins from Cuba, the Dominican Republic, Mexico, Puerto Rico, and Central and South America. The sample design and cohort selection have been described previously.^5^

Eligible individuals visited the field center, informed consent was obtained, and study assessments were completed. From March 2008 to June 2011, 16,415 Hispanics and Latinos were enrolled. Baseline data included medical history, physical examination and assessments of acculturation to the U.S. culture, health behaviors (including diet and physical activity), and health care access.^6^ HCHS/SOL protocols were approved by the Institutional Review Boards at all field, reading, and coordinating centers.

### Dietary data collection

Diet was assessed using two 24-h recalls and a food propensity questionnaire.^7^ One recall was conducted in person at baseline and the other through telephone within 30 days. Interviews were conducted as participants preferred in Spanish (80% of participants) or English (20% of participants), employing the Nutrition Data System for Research (NDSR) software (version 11).^8^ Participant-completed food amounts booklets were distributed to estimate portion sizes at subsequent interviews. As per protocol, nearly all participants (99%) provided the first recall and 88% provided both.

### Measures of U.S. acculturation

Participants completed an abbreviated version of Marin's Short Acculturation Scale for Hispanics (SASH).^9^ Two media items were eliminated from the original questionnaire. Psychometric analysis identified two components: language preference (SASH language) and ethnic social relationships (SASH social). Internal consistency for each component was estimated (α=0.92 [language] and 0.73 [social]).^10^ Factor analysis supported validity of the two-factor solution and configured invariance across Spanish and English versions. HCHS/SOL also includes traditional proxy measures of U.S. acculturation (time in the United States [50 states only], born in the United States [50 states only], and language preference).

### Covariates

Annual household income, Hispanic/Latino heritage, age (years), sex, employment status, marital status, educational attainment, and cigarette use were self-reported. Body mass index (BMI) was calculated using measured height and weight and classified into weight status groups (underweight BMI <18.5, normal weight BMI 18.5–24.9, overweight BMI 25–30, and obese BMI ≥30 kg/m^2^).^11^ Blood pressure was measured, and blood samples were collected and analyzed to check values of various biomarkers; both fasting and post-oral glucose challenge blood samples were collected. Participants presented all medications used in the past 4 weeks, and this information was recorded. High cholesterol, diabetes, and hypertension were defined based on a combination of measured biomarker/blood pressure values and medication use.^12–14^

### Statistical methods

Exclusion criteria were age ≥74 years (*n*=9), missing Hispanic/Latino heritage (*n*=87), other/mixed Hispanic/Latino heritages (*n*=503), no dietary recalls (*n*=37), unreliable recalls, or energy intakes below the first percentile or above the 99th percentile by sex (*n*=146), resulting in a final sample size of 15,633.

Using dietary recalls, we calculated HEI scores using the NDSR documentation guide by counting food group servings or nutrient intake.^15^ HEI 2010 has 12 components: 9 reflect intake adequacy of total fruit, whole fruit, total vegetables, greens and beans, whole grains, dairy, total protein foods, seafood and plant proteins, and fatty acids, and 3 moderation components reflect limited intake of refined grains, sodium, and empty calories (calories from solid fats, alcohol (threshold >13 g/1000 kcal), and added sugars).^2^ The score for adequacy components increases with increased consumption, while the score for moderation components increases with decreased consumption. Score standards are density based (i.e., servings/calories). For all components, a higher score indicates closer adherence to dietary guidance and also suggests a healthier diet; total HEI score ranges from 0 to 100.

Criteria for defining episodically consumed food groups were consistent with previous research using this sample.^7^ Distribution of these 12 components and total energy intake were estimated jointly using the multivariate Markov Chain Monte Carlo National Cancer Institute (NCI) method,^16,17^ which corrects for high intraindividual variation, and accounts for correlation between different dietary components. Specifically, a multivariate nonlinear mixed model considered intake among all components and total energy and the consumption probability for episodically consumed food groups. Details of the measurement error model are described elsewhere.^17^ All parts of the model adjusted for sex, age, Hispanic/Latino heritage, field center, weekend (including Friday), self-reported intake amount (more, same, or less than usual amount), and dietary recall sequence (first or second). Dietary intake amounts were transformed using a log transformation for fatty acids and a Box-Cox transformation with parameter 0.25 for all other food groups and energy, and centered and scaled to mean 0 and variance 2. After fitting the model, a multivariate Monte Carlo distribution of the 12-component usual intake and total energy was generated by randomly drawing 100 sets of intake for each participant conditional on covariates, creating a “pseudo-population,” and the component scores and total score were calculated for this pseudo-population.^17,18^ This pseudo-population represented the joint distribution of usual intake for the component scores and total score, and was used to calculate all statistics. Standard errors (SEs) were estimated by bootstrapping to account for the complex survey design.^19^

Means and SEs for the total score and each component score were calculated for the full sample and by Hispanic/Latino heritage, sex, age group, and U.S. acculturation measures; the percent meeting recommendations for each dietary component were calculated for the full sample and by Hispanic/Latino heritage. When calculating statistics by subpopulations, the measurement error model was estimated separately, allowing all model parameters to differ by subpopulation. The joint distribution of dietary components was adjusted to the full sample mean age (except for age subpopulations) and proportion of male (except for sex subpopulations).

Finally, we used complex survey linear regression to examine the correlates of HEI 2010 scores. Only for this analysis, we used average intake from the 24-h recalls to calculate the HEI scores rather than NCI-predicted intakes, because assessing the statistical association between usual intake of multivariate diet measures and other variables is still under development.^20^ We selected correlates using a step-wise backward model, considering age, sex, BMI group, cigarette use, physical activity level, education, income, employment, marital status, Hispanic/Latino heritage, nativity, years in the United States (50 states only), language of preference, SASH language score, SASH social score, field center, and the presence of three medical conditions (high total cholesterol, diabetes, and hypertension). Variables were dropped one at a time beginning with the least significant, until all covariates remaining were significant (*p*=0.1). Field center and Hispanic/Latino heritage were forced to stay in the model.

All analyses, conducted from December 2017 to August 2018, accounted for the complex survey design and sampling weights by using SAS (version 9.3) survey procedures or SAS-callable SUDAAN (version 10) software package (RTI International). To account for complex survey design, while handling the iterative process of backward selection, we used a macro developed by Wang and Shin.^21^

## Results

Mexicans are the largest heritage group represented in HCHS/SOL (39%) followed by Cubans (21%) and Puerto Ricans (17%) (Supplementary Table S1). Each of the other Hispanic heritage groups ranges from 5% to 10% in size. Half were female and 21% were born outside of 50 U.S. states. Employment status varied by heritage, with Central and South Americans and Mexicans having higher rates of employment compared to Puerto Ricans. South Americans and Cubans had similar distribution of educational status with roughly half completing more than high school, with other groups closer to one-third. Mexicans reported the highest proportion married or cohabitating (60%), while Puerto Ricans had the lowest (34%). The overall prevalence of obesity was 39%, also varying by heritage, with South Americans having the lowest and Puerto Ricans the highest. Medical conditions of diabetes, hypertension, and hypercholesterolemia were common among HCHS/SOL participants, with an overall prevalence of 14.8%, 22.1%, and 42.4%, respectively.

The mean HEI total score and each component score for the population and by heritage, age- and sex-adjusted, appears in Table 1. The overall HEI mean, 63.8 (maximum 100), varied across groups from a high of 71 (Mexicans) to a low of 56 (Puerto Ricans). Across HEI component adherence, 87% of all participants had the maximum score for total protein foods with little variability across the Hispanic/Latino heritage groups (Table 2). HEI components with maximum scores, indicating that the recommendation has been achieved, reported by one-fourth to more than three-fourths of adults included whole fruits, greens and beans, and seafood and plant proteins; only two groups deviated from this pattern: Cubans and Puerto Ricans had lower adherence scores for whole fruits and greens and beans (Table 2). Among Mexicans, 45% adhered to the whole grain and 25% adhered to the refined grain recommendation; other groups had much lower adherence rates (0.8–8.3% and 5.1–14.8% respectively), with Puerto Ricans having the lowest rates. Except for adults of Cuban heritage, all other heritages had <10% adhering to recommendations for fatty acids (PUFAs+MUFAs)/SFAs >2.5). Sodium was the HEI component with the lowest adherence to the recommendations, with all groups below 2% (Table 2).

**Table 1.** Healthy Eating Index Total and Component Mean Scores by Hispanic/Latino Heritage, HCHS/SOL (2008–2011)^a^

HEI component	Range	Overall (*n*=15633)	Mexicans (*n*=6419)	Central Americans (*n*=1707)	Cubans (*n*=2318)	Dominicans (*n*=1453)	Puerto Ricans (*n*=2672)	South Americans (*n*=1064)
HEI 1: Total fruit	0–5	2.80 (0.06)	2.91 (0.11)	2.93 (0.38)	2.47 (0.17)	3.86 (0.19)	2.38 (0.12)	3.50 (0.14)
HEI 2: Whole fruit	0–5	3.05 (0.00)	3.95 (0.01)	3.39 (0.42)	2.69 (0.05)	4.63 (0.22)	2.50 (0.11)	3.90 (0.23)
HEI 3: Total vegetables	0–5	3.27 (0.02)	3.48 (0.04)	3.19 (0.35)	3.39 (0.02)	3.21 (0.07)	2.77 (0.01)	3.48 (0.05)
HEI 4: Greens and beans	0–5	3.17 (0.14)	3.41 (0.25)	3.39 (1.08)	3.23 (0.07)	3.08 (0.03)	2.64 (0.13)	3.38 (0.13)
HEI 5: Whole grains	0–10	4.81 (0.02)	8.28 (0.02)	4.60 (3.76)	1.72 (0.02)	2.28 (0.13)	2.83 (0.26)	3.52 (0.43)
HEI 6: Dairy	0–10	5.78 (0.01)	5.96 (0.00)	5.25 (1.21)	5.39 (0.26)	5.62 (0.17)	6.45 (0.17)	5.69 (0.02)
HEI 7: Total protein foods	0–5	4.92 (0.02)	4.94 (0.03)	4.96 (0.00)	4.97 (0.02)	4.98 (0.01)	4.87 (0.01)	4.92 (0.05)
HEI 8: Seafood and plant proteins	0–5	3.95 (0.04)	4.18 (0.16)	4.33 (0.51)	4.01 (0.13)	4.30 (0.12)	3.41 (0.04)	3.93 (0.05)
HEI 9: Fatty acids	0–10	5.94 (0.03)	5.85 (0.13)	6.81 (0.76)	6.47 (0.06)	6.74 (0.19)	4.80 (0.07)	5.72 (0.00)
HEI 10: Refined grains	0–10	6.41 (0.03)	7.62 (0.04)	6.19 (1.13)	5.82 (0.09)	6.28 (0.26)	5.48 (0.06)	5.97 (0.05)
HEI 11: Sodium	0–10	3.78 (0.02)	4.84 (0.04)	3.41 (0.32)	2.25 (0.13)	3.25 (0.64)	3.89 (0.21)	2.79 (0.12)
HEI 12: Empty calories^b^	0–20	15.93 (0.10)	15.72 (0.11)	16.25 (0.57)	16.79 (0.33)	17.70 (0.08)	14.44 (0.11)	16.53 (0.19)
Total HEI score	0–100	63.82 (0.39)	71.14 (0.86)	64.69 (4.86)	59.20 (0.26)	65.92 (1.21)	56.45 (0.06)	63.35 (0.38)

^a^ Adjusted by age and sex (mean age: 41.30, % male: 47.76). HEI components calculated based on multivariate NCI method (see statistical methods for details). Higher total score is indicative of a healthier diet.

^b^ Calories from solid fats, alcohol (threshold >13 g/1000 kcal), and added sugars.

HCHS/SOL, Hispanic Community Health Study/Study of Latinos; HEI, Healthy Eating Index; NCI, National Cancer Institute.

**Table 2.** Percent of Hispanic/Latino Adults with a Maximum Healthy Eating Index Component Score by Hispanic/Latino Heritage, HCHS/SOL (2008–2011)^a^

HEI component	Standard of maximum score	Overall (*n*=15633)	Mexicans (*n*=6419)	Central Americans (*n*=1707)	Cubans (*n*=2318)	Dominicans (*n*=1453)	Puerto Ricans (*n*=2672)	South Americans (*n*=1064)
Adequacy								
HEI 1: Total fruit	≥0.8 cup equiv/1000 kcal	17.64 (0.73)	15.51 (2.18)	22.66 (0.05)	11.09 (1.28)	42.18 (1.99)	7.29 (2.14)	28.01 (2.65)
HEI 2: Whole fruit	≥0.4 cup equiv/1000 kcal	32.03 (0.78)	45.78 (0.10)	38.39 (11.96)	21.49 (0.99)	79.20 (5.46)	18.51 (2.22)	46.87 (9.20)
HEI 3: Total vegetables	≥1.1 cup equiv/1000 kcal	15.43 (0.55)	16.89 (0.27)	12.04 (7.69)	13.43 (2.02)	11.65 (1.29)	5.55 (0.38)	18.61 (0.27)
HEI 4: Greens and beans	≥0.2 cup equiv/1000 kcal	26.05 (1.33)	29.80 (3.49)	25.32 (54.50)	23.41 (0.88)	26.13 (2.56)	14.15 (0.48)	27.16 (5.76)
HEI 5: Whole grains	≥1.5 oz equiv/1000 kcal	18.41 (0.12)	45.19 (0.52)	8.32 (64.61)	1.50 (0.47)	1.02 (0.32)	0.81 (0.69)	3.32 (0.82)
HEI 6: Dairy	≥1.3 cup equiv/1000 kcal	16.10 (0.05)	13.94 (1.53)	9.87 (5.96)	15.25 (1.09)	10.52 (3.44)	19.29 (1.51)	12.09 (2.10)
HEI 7: Total protein foods	≥2.5 oz equiv/1000 kcal	87.26 (1.87)	89.05 (3.75)	91.03 (1.00)	93.16 (3.03)	94.86 (2.17)	81.07 (1.62)	87.46 (6.02)
HEI 8: Seafood and plant proteins	≥0.8 oz equiv/1000 kcal	45.92 (0.61)	53.78 (5.20)	54.49 (32.14)	39.78 (3.91)	56.80 (6.70)	28.26 (0.23)	45.18 (6.63)
HEI 9: Fatty acids	(PUFAs+MUFAs)/SFAs >2.5	7.40 (0.25)	3.54 (0.24)	9.50 (14.18)	16.02 (0.46)	7.65 (0.75)	2.06 (0.50)	3.26 (0.22)
Moderation								
HEI 10: Refined grains	≤1.8 oz equiv/1000 kcal	18.64 (0.09)	24.96 (0.26)	7.30 (3.25)	7.98 (0.94)	12.78 (0.01)	5.18 (0.00)	14.77 (3.00)
HEI 11: Sodium	≤1.1 g/1000 kcal	0.79 (0.13)	1.71 (0.65)	0.23 (0.07)	0.20 (0.00)	0.05 (0.04)	0.26 (0.01)	0.04 (0.01)
HEI 12: Empty calories^b^	≤19% of energy	14.42 (0.23)	9.15 (0.37)	15.21 (6.44)	18.21 (0.74)	30.83 (0.84)	7.51 (1.10)	17.24 (0.20)

^a^ Adjusted by age and sex accordingly (mean age: 41.30, % male: 47.76). HEI components calculated based on multivariate NCI method (see statistical methods for details).

^b^ Calories from solid fats, alcohol (threshold >13 g/1000 kcal), and added sugars.

MUFA, mono unsaturated fatty acid; PUFA, poly unsaturated fatty acid; SFA, saturated fatty acid.

HEI total and individual mean component scores also varied by sex and age (Supplementary Table S2). Higher scores were reported by women compared to men, and individuals >30 years compared to those 18–30 years of age. The pattern of scores for total fruit and whole fruit is similar. In addition, women reported a one-unit higher score for dairy; participants >30 years of age reported a one-unit higher score for seafood and plant proteins, and two-units higher for empty calories. Otherwise, mean component scores varied by a smaller magnitude across all sex and age groupings. Table 3 displays the HEI total and component mean scores by four measures of acculturation to United States: SASH social and language scores, being born in the United States (50 states only), and language preference. We see greater differentiation by more traditional measures of acculturation and the SASH language score than the SASH ethnic social relationship subscale. Individuals with less U.S. acculturation (those not born in the United States (50 states only), prefer to speak Spanish, or have low SASH language score) have higher HEI scores. Component scores that appear higher for individuals with less U.S. acculturation include those related to fruit and vegetable intake, and seafood and plant proteins.

**Table 3.** Healthy Eating Index Total and Component Mean Scores for Hispanic/Latino Adults by Measures of Acculturation, HCHS/SOL (2008–2011)^a^

HEI component	SASH social score		SASH language score		United States born (50 states only)		Language preference	
	Low: score ≤2 (*n*=6901)	High: score >2 (*n*=8112)	Low: score ≤2 (*n*=10447)	High: score >2 (*n*=5155)	Yes (*n*=2545)	No (*n*=13087)	Spanish (*n*=12662)	English (*n*=2971)
HEI 1: Total fruit	2.79 (0.12)	2.81 (0.01)	2.99 (0.08)	2.41 (0.03)	1.96 (0.09)	2.95 (0.06)	2.95 (0.00)	2.17 (0.02)
HEI 2: Whole fruit	3.31 (0.44)	3.13 (0.16)	3.88 (0.01)	2.74 (0.32)	2.05 (0.06)	3.24 (0.46)	3.83 (0.35)	2.52 (0.18)
HEI 3: Total vegetables	3.29 (0.04)	3.22 (0.03)	3.42 (0.02)	3.00 (<0.01)	2.87 (0.01)	3.36 (0.01)	3.41 (0.02)	2.79 (0.03)
HEI 4: Greens and beans	3.20 (0.06)	3.15 (0.26)	3.32 (0.12)	2.91 (0.13)	2.79 (0.26)	3.26 (0.07)	3.30 (0.15)	2.66 (<0.01)
HEI 5: Whole grains	4.92 (0.12)	4.77 (0.06)	4.92 (0.07)	4.51 (0.06)	4.31 (0.00)	4.85 (0.04)	4.91 (0.07)	4.24 (0.14)
HEI 6: Dairy	5.56 (0.07)	5.98 (0.08)	5.67 (0.12)	5.86 (0.16)	5.84 (0.25)	5.75 (0.05)	5.75 (0.09)	5.85 (0.27)
HEI 7: Total protein foods	4.94 (0.02)	4.92 (0.01)	4.95 (0.02)	4.90 (0.01)	4.93 (0.01)	4.93 (0.02)	4.93 (0.03)	4.92 (0.01)
HEI 8: Seafood and plant proteins	4.22 (0.02)	3.80 (0.02)	4.18 (0.09)	3.62 (0.14)	3.31 (0.27)	4.11 (0.09)	4.08 (0.10)	3.57 (0.19)
HEI 9: Fatty acids	6.19 (0.07)	5.72 (0.05)	6.11 (0.13)	5.72 (0.16)	5.45 (0.21)	6.05 (0.07)	6.09 (0.08)	5.45 (0.15)
HEI 10: Refined grains	6.57 (0.05)	6.36 (0.01)	6.62 (0.08)	5.98 (0.07)	5.72 (0.32)	6.54 (0.11)	6.59 (0.09)	5.63 (0.32)
HEI 11: Sodium	3.60 (0.10)	3.98 (0.03)	3.59 (0.09)	4.05 (0.08)	4.01 (0.11)	3.69 (0.06)	3.66 (0.06)	4.16 (0.18)
HEI 12: Empty calories^b^	16.46 (0.14)	15.46 (0.03)	16.56 (0.15)	14.68 (0.06)	13.66 (0.21)	16.35 (0.14)	16.38 (0.15)	14.06 (0.19)
Total HEI score	65.05 (0.20)	63.30 (0.30)	66.19 (0.40)	60.39 (0.38)	56.90 (0.25)	65.06 (0.03)	65.88 (0.74)	58.02 (0.11)

^a^ Adjusted by age and sex (mean age: 41.30, % male: 47.76). HEI components calculated based on multivariate NCI method (see statistical methods for details). Higher total score is indicative of a healthier diet.

^b^ Calories from solid fats, alcohol (threshold >13 g/1000 kcal), and added sugars.

SASH, Short Acculturation Scale for Hispanics.

Our model of adherence to the recommendations (shown in Table 4) explained 22% of the variance. Characteristics associated with better adherence to dietary recommendations included increasing age, greater than high school education, increasing household income beyond $10,000, being female, Spanish language preference, being born outside the 50 U.S. states, and having diabetes. In addition, Mexican heritage was associated with the highest adherence relative to the other groups. Being of normal weight was associated with the higher adherence relative to individuals with obesity and never and former smokers were more adherent than current smokers. Finally, those with any level of physical activity were more adherent than those physically inactive.

**Table 4.** Regression Coefficients for Model of Factors for Adherence to the Healthy Eating Index, HCHS/SOL (2008–2011)

Parameter	Estimate	95% lower confidence limit	95% upper confidence limit
Intercept	44.00	41.43	46.57
Age, years	0.20	0.17	0.23
Gender, female	2.72	2.04	3.39
Hispanic heritage			
Central American	−3.98	−5.37	−2.58
Cuban	−7.77	−9.40	−6.15
Dominican	−2.07	−3.45	−0.68
Mexican	REF	—	—
Puerto Rican	−7.65	−9.07	−6.23
South American	−6.19	−7.66	−4.71
Field center			
Bronx	REF	—	—
Chicago	5.24	4.17	6.30
Miami	1.86	0.38	3.34
San Diego	3.08	1.71	4.44
Education			
No high school/GED	REF	—	—
At most high school/GED	0.31	−0.47	1.10
Greater than high school/GED	1.22	0.41	2.04
Household income			
<$10,000	REF	—	—
$10,001–$20,000	1.05	0.11	1.99
$20,001–$40,000	1.16	0.17	2.14
$40,001–$75,000	1.83	0.62	3.03
>$75,000	3.95	1.74	6.16
Weight status category			
Underweight (BMI <18.5 kg/m^2^)	−1.19	−3.69	1.32
Normal (BMI 18.5–24.9 kg/m^2^)	1.19	0.36	2.02
Overweight (BMI 25–29.9 kg/m^2^)	0.55	−0.14	1.24
Obese (BMI ≥30 kg/m^2^)	REF	—	—
Cigarette use			
Never	3.22	2.37	4.07
Former	2.23	1.05	3.41
Current	REF	—	—
Activity level per 2008 guidelines			
Inactive	REF	—	—
Low activity	1.38	0.29	2.47
Medium activity	1.71	0.75	2.67
High activity	1.62	0.86	2.38
Language preference, Spanish	1.71	0.36	3.06
Language acculturation (SASH subscale)	−0.74	−1.20	−0.28
United States born (50 states only)	−3.18	−4.53	−1.84
Diabetes	2.44	1.53	3.34
Hypertension	−0.74	−1.61	0.12

BMI, body mass index; GED, general equivalency diploma.

## Discussion

Employing the HEI, an indicator of diet quality based on adherence to the DGA, not only individuals with Mexican heritage^22,23^ but also those of Dominican and Central American heritage, had better overall diet quality compared to other Hispanic/Latino heritage groups. In addition, the proportion with a maximum score for the HEI components varied across heritage groups; Mexicans had a higher proportion with maximum scores for whole grains and refined grains, whereas Dominicans had a higher proportion for total fruit, whole fruits, and moderation in the consumption of empty calories, but a lower proportion for whole grains. In contrast, Central Americans and Cubans had a higher proportion of maximum scores for fatty acids. This variability illustrates the importance of differentiating heritage groups since these nuances are lost when all Hispanics/Latinos are combined, making the assumption that their food intake patterns are similar. Our results show that individuals from Puerto Rico had the lowest HEI scores, with 10 of 12 components having the lowest proportion of individuals meeting the maximum score (every component except dairy and sodium). This examination allows us to identify areas where necessary improvements are unique to the food intake patterns of each country of origin. These areas reflect important intervention avenues for health care providers and nutritionists toward improving health outcomes. The standard one-size message does not fit all Hispanics/Latinos. These data help fill gaps identified in the Scientific Report of the Dietary Guidelines Advisory Committee that reports data only on “Mexicans and other Hispanics” to interpret how individuals in the United States meet 2010 Dietary Guidelines recommendations.

Among Hispanics/Latinos in HCHS/SOL, as for all Americans, women had higher HEI scores compared to men, and scores increased with age.^2,3,18,22,23^ The overall mean score for this cohort, 63.8, although suggesting need for improvement among Hispanics/Latinos, reflects higher diet quality compared with NHANES findings among non-Hispanic whites (56.2) and non-Hispanic blacks (51.0).^23^ Other measures of diet quality, such as the Alternative HEI, have demonstrated similar interpretations within the HCHS/SOL population^24^; however, the HEI is the accepted measure used by the Dietary Guidelines committee that reflects adherence to the U.S. policy.^1^

At baseline, several measures of U.S. acculturation allowed us to explore differences in diet quality, which help explain the association between diet and health outcomes among immigrants. While the SASH scale (two subscales) operationalizes acculturation beyond the traditional measures, the ethnic social relationship subscale did not help to differentiate between individuals with higher or lower scores of dietary quality. Our model of variables associated with HEI score included U.S. acculturation measures (i.e., language acculturation, language preference, and being born in the United States [50 states only]), each of which remained significant. Overall, these results are congruent with others, demonstrating that less acculturation, or the preservation of one's cultural traditions, is associated with better quality diet.^25,26^

Additional findings in this study demonstrate support for lifestyle behaviors, such as not being a current smoker, and/or having diabetes, as being associated with greater adherence to the Dietary Guidelines. Other research supports that nonsmokers have healthier diets,^27,28^ and those who acknowledge having diabetes are more likely to watch what they eat, resulting in better quality diets; indeed, this is often the first line of treatment.^27^ Previous analysis in this cohort suggested that individuals with knowledge about their diabetes status and whose diabetes was controlled (hemoglobin A1c <7%) had better quality diets than those with pre-diabetes.^29^ On the other hand, we cannot rule out the possibility of social desirability bias in the reporting of diet among this subgroup.^30^

This study has several limitations. First, results have limited generalizability specific to the four communities sampled. Nevertheless, these cities are among the largest in concentration of Hispanics/Latinos in urban areas. Regardless, HCHS/SOL's hybrid design, employing probability sampling within pre-selected regions, is more rigorous than a simple convenience sample typical of most epidemiological cohort studies. Second, as with most large cohort surveys, response rates are lower than desired, which could result in bias if adjustments accounting for systematic differences between responders and nonresponders are not made. In an attempt to overcome this issue, we applied a statistical adjustment protocol to reduce the potential bias of estimates due to nonparticipation. This approach is consistent with weighting strategies for all major health surveys utilizing probability sampling (e.g., NHANES, National Health Interview Survey [NHIS], and Medical Expenditure Survey [MEPS]). Third, inevitable measurement error inherent in self-reported diet means usual intake may be misreported. In an ancillary study, we assessed measurement error of self-reported energy, protein, sodium, and potassium intake using doubly labeled water, and urinary nitrogen, sodium, and potassium, and found underestimation of these nutrients was partly related to BMI and ethnicity among other factors in HCHS/SOL.^31^ Age-adjusted sodium intake based on urinary sodium was higher than the recommendation of 2300 mg/day and was almost twice as high as the recommendation among Cubans.^32^ Finally, the analyses conducted herein were cross-sectional in nature, and thus causality cannot be inferred; we can only identify variables associated with dietary quality.

Despite these limitations, the study has many strengths, including the use of two interviewer-administered 24-h recalls assessing dietary intake. This allowed us to apply NCI methodology for estimating usual intake of multivariate diet,^17^ allowing person-specific random effects in the model. This accommodates many complex aspects of estimating usual intake, including high day-to-day variability, zero inflation of intake reported by a single 24-h recall since some foods are not consumed every day, and correlation between different dietary components.^17^ Second, diet quality was characterized by the HEI, a validated tool that has been used to link diet and health outcomes.^18^ By assessing overall diet quality, we examined the effect of all nutrients provided by the foods consumed and how they interact, perhaps synergistically, to influence metabolic health and health profile. Data from other studies using this approach report that such indices are more strongly associated with mortality than any one component of diet alone.^33^ Third, this large cohort of Hispanics/Latinos with various heritage groups allowed us to examine differences, never previously explored, among these groups. Finally, we used validated acculturation measures for assessing cultural orientation and ethnic identity, alongside commonly used single-item measures (e.g., nativity) that are proxies for U.S. acculturation.

## Conclusion

In summary, this analysis of diet quality within the HCHS/SOL cohort demonstrates that Hispanics/Latinos from any heritage can improve upon their eating habits to more closely align with the DGA, with sodium consumption and improved fatty acids ratio being areas where much improvement is needed. Other target areas for improvement differ by many factors, including Hispanic/Latino heritage. Thus, interventions designed to improve upon diet, a lifestyle behavior that has been repeatedly associated with cardiometabolic outcomes and overall mortality,^4,33^ should be tailored specifically to the heritage group from which Hispanics/Latinos originate, as well as by sex, age, physical activity behavior, language preference, and smoking habit.

## Supplementary Material

Supplemental data

Supplemental data

## Abbreviations Used

CI: confidence interval
DGA: Dietary Guidelines for Americans
GED: general equivalency diploma
HCHS/SOL: Hispanic Community Health Study/Study of Latinos
HEI: Healthy Eating Index
MEPS: Medical Expenditure Panel Survey
MUFA: mono unsaturated fatty acid
NCI: National Cancer Institute
NDSR: Nutrition Data System for Research
NHANES: National Health and Nutrition Evaluation Study
NHIS: National Health Interview Survey
PUFA: poly unsaturated fatty acid
SASH: Short Acculturation Scale for Hispanics
SE: standard error
SFA: saturated fatty acid
