# Supplementary material for: How Well Do U.S. Hispanics Adhere to the Dietary Guidelines for Americans? Results from the Hispanic Community Health Study/Study of Latinos
Source: Health Equity. 2019 Jul 11;3(1):319–27. doi: 10.1089/heq.2018.0105 (PMC6643200; doi:10.1089/heq.2018.0105)
Supplement: Supplemental data [file Suppl_TableS1.pdf]

# Supplementary Data

**Supplementary Table S1. Sociodemographic, Acculturation, Behavior, and Medical Characteristics by Hispanic/Latino Heritage ($n = 15{,}633$), HCHS/SOL (2008–2011)**

| Characteristic | Overall % (95% CI) ($n = 15633$) | Mexican % (95% CI) ($n = 6419$) | Central American % (95% CI) ($n = 1707$) | Cuban % (95% CI) ($n = 2318$) | Dominican % (95% CI) ($n = 1453$) | Puerto Rican % (95% CI) ($n = 2672$) | South American % (95% CI) ($n = 1064$) |
|---|---|---|---|---|---|---|---|
| Age (years) | 41.3 (40.8–41.8) | 38.5 (37.8–39.2) | 39.7 (38.8–40.7) | 46.6 (45.6–47.6) | 39.1 (37.7–40.5) | 43.0 (42.0–44.0) | 42.3 (40.8–43.8) |
| Female (%) | 52.2 (51.2–53.3) | 53.2 (51.3–55.1) | 52.4 (49.0–55.7) | 47.9 (45.9–50.0) | 60.4 (56.7–64.0) | 49.7 (46.9–52.5) | 54.3 (50.3–58.3) |
| Education (%) | | | | | | | |
| No high school or GED | 32.7 (31.2–34.1) | 36.1 (33.5–38.8) | 38.2 (35.0–41.4) | 21.9 (19.9–24.1) | 36.8 (33.4–40.3) | 36.3 (33.2–39.6) | 21.5 (18.1–25.4) |
| At most a high school or GED | 28.5 (27.4–29.6) | 29.8 (27.9–31.8) | 26.5 (23.8–29.5) | 29.6 (27.0–32.4) | 23.9 (20.4–27.7) | 28.0 (25.6–30.5) | 27.5 (24.0–31.2) |
| Greater than high school or GED | 38.9 (37.3–40.5) | 34.0 (31.0–37.2) | 35.3 (32.1–38.6) | 48.4 (45.6–51.3) | 39.4 (36.0–42.8) | 35.7 (32.6–39.0) | 51.0 (46.8–55.1) |
| Yearly household income (%) | | | | | | | |
| <$10,000 | 14.6 (13.6–15.8) | 10.4 (9.0–12.0) | 15.6 (13.0–18.5) | 19.1 (16.8–21.7) | 16.2 (13.6–19.1) | 20.0 (17.5–22.7) | 10.2 (8.1–12.8) |
| $10,000–$20,000 | 32.0 (30.5–33.6) | 28.5 (25.9–31.4) | 37.9 (34.1–41.8) | 36.0 (33.2–38.9) | 37.2 (32.9–41.7) | 29.6 (26.8–32.6) | 33.4 (29.7–37.4) |
| $20,000–$40,000 | 33.3 (32.1–34.7) | 36.6 (34.4–38.9) | 33.1 (29.6–36.7) | 30.4 (27.7–33.3) | 33.2 (29.4–37.2) | 27.7 (24.6–31.1) | 37.4 (33.4–41.5) |
| $40,000–$75,000 | 14.3 (13.2–15.5) | 17.2 (15.3–19.3) | 11.1 (8.7–14.1) | 9.9 (8.3–11.8) | 10.5 (8.4–13.0) | 15.9 (13.8–18.4) | 14.0 (11.2–17.3) |
| >$75,000 | 5.7 (4.6–7.0) | 7.2 (5.2–10.0) | 2.4 (1.5–3.8) | 4.5 (3.3–6.2) | 3.0 (1.7–5.2) | 6.7 (5.3–8.5) | 4.9 (3.2–7.4) |
| Spanish preference (%) | 76.1 (74.3–77.8) | 78.0 (75.9–80.1) | 87.8 (84.5–90.5) | 92.7 (90.8–94.2) | 75.9 (70.8–80.4) | 41.3 (37.9–44.9) | 89.7 (86.6–92.1) |
| Language acculturation | 2.1 (2.0–2.1) | 2.1 (2.0–2.1) | 1.7 (1.6–1.8) | 1.6 (1.5–1.6) | 2.0 (1.9–2.1) | 3.1 (3.0–3.2) | 1.8 (1.7–1.9) |
| Social acculturation | 2.2 (2.2–2.3) | 2.2 (2.2–2.2) | 2.1 (2.0–2.2) | 2.0 (2.0–2.0) | 2.3 (2.3–2.4) | 2.5 (2.5–2.5) | 2.3 (2.2–2.3) |
| Current smoking (%) | 21.1 (20.0–22.2) | 17.8 (16.1–19.7) | 14.7 (12.5–17.2) | 26.0 (23.6–28.6) | 12.0 (9.2–15.4) | 33.2 (30.5–36.0) | 13.3 (10.6–16.6) |
| BMI (%) | | | | | | | |
| Under/normal (BMI <25 kg/m$^2$) | 22.9 (21.9–24.0) | 22.3 (20.7–24.0) | 24.2 (21.3–27.4) | 24.8 (22.5–27.2) | 22.1 (19.3–25.1) | 20.2 (17.8–22.8) | 28.7 (24.5–33.3) |
| Overweight (BMI 25–29.9 kg/m$^2$) | 37.6 (36.4–38.8) | 39.2 (36.9–41.5) | 38.6 (35.2–42.1) | 37.0 (34.7–39.5) | 37.6 (34.2–41.2) | 32.7 (29.9–35.6) | 41.7 (37.7–45.9) |
| Obese (BMI ≥30 kg/m$^2$) | 39.5 (38.1–40.8) | 38.5 (36.0–41.0) | 37.1 (34.3–40.0) | 38.2 (35.8–40.7) | 40.3 (36.6–44.2) | 47.1 (44.0–50.3) | 29.6 (26.0–33.4) |
| Study center (%) | | | | | | | |
| Bronx | 28.2 (25.5–31.1) | 8.2 (6.2–10.8) | 19.6 (15.2–24.9) | 1.5 (1.0–2.4) | 94.5 (91.4–96.5) | 72.2 (68.2–75.9) | 24.8 (19.6–31.0) |
| Chicago | 16.1 (14.3–18.2) | 26.2 (22.7–30.1) | 14.4 (11.2–18.3) | 0.8 (0.3–1.9) | 0.8 (0.5–1.4) | 20.6 (17.4–24.3) | 20.6 (16.0–26.1) |
| Miami | 29.4 (25.3–33.8) | 1.1 (0.7–1.8) | 62.1 (54.9–68.8) | 97.3 (95.8–98.3) | 4.4 (2.5–7.4) | 4.6 (3.2–6.4) | 50.2 (43.1–57.3) |
| San Diego | 26.3 (23.0–29.8) | 64.4 (59.9–68.7) | 3.9 (2.6–5.9) | 0.4 (0.1–1.4) | 0.4 (0.1–1.4) | 2.6 (1.5–4.5) | 4.3 (2.3–8.2) |
| Born in 50 U.S. states (%) | 21.3 (19.9–22.9) | 23.7 (21.7–25.7) | 7.4 (5.4–10.0) | 7.1 (5.5–9.2) | 16.9 (13.3–21.2) | 47.8 (44.7–50.9) | 5.7 (4.1–7.8) |
| Born in United States or U.S. territory (%) | 29.6 (27.7–31.6) | 23.7 (21.7–25.8) | 7.8 (5.8–10.4) | 7.2 (5.6–9.2) | 17.1 (13.5–21.3) | 97.0 (95.5–98.0) | 5.9 (4.3–8.1) |
| Years lived in mainland United States | 20.1 (19.5–20.8) | 20.0 (19.3–20.6) | 14.9 (14.0–15.8) | 14.0 (12.7–15.2) | 17.9 (17.0–18.9) | 33.6 (32.5–34.7) | 14.6 (13.5–15.7) |
| Employment (%) | | | | | | | |
| Retired and not employed | 8.3 (7.6–9.0) | 4.5 (3.7–5.3) | 4.3 (3.3–5.6) | 11.4 (9.8–13.4) | 8.0 (6.4–9.9) | 16.3 (14.0–18.9) | 5.6 (4.1–7.6) |
| Not retired and not employed | 41.0 (39.6–42.5) | 38.5 (36.4–40.6) | 36.2 (33.1–39.3) | 47.4 (44.6–50.1) | 42.2 (38.1–46.4) | 43.6 (40.4–46.9) | 31.3 (27.9–34.9) |
| Employed part time | 16.9 (16.0–17.8) | 19.8 (18.2–21.4) | 23.1 (20.6–25.8) | 11.3 (10.0–12.8) | 16.9 (14.3–20.0) | 11.9 (10.0–14.0) | 23.7 (20.7–26.9) |
| Employed full time | 33.8 (32.5–35.1) | 37.3 (35.3–39.3) | 36.4 (33.5–39.4) | 29.9 (27.6–32.3) | 32.9 (29.4–36.6) | 28.2 (25.5–31.1) | 39.5 (35.8–43.3) |
| Marital status (%) | | | | | | | |
| Single | 33.8 (32.5–35.1) | 28.2 (26.1–30.3) | 39.3 (36.4–42.3) | 25.7 (23.3–28.3) | 47.7 (44.1–51.4) | 47.0 (44.0–50.1) | 30.1 (26.3–34.2) |
| Married/with partner | 49.7 (48.2–51.3) | 59.7 (57.2–62.0) | 46.1 (42.9–49.2) | 51.6 (48.6–54.5) | 37.1 (33.7–40.6) | 33.6 (30.7–36.7) | 49.6 (45.3–53.9) |
| Separated/divorced/ widowed | 16.4 (15.5–17.4) | 12.2 (10.9–13.5) | 14.6 (12.8–16.6) | 22.7 (20.5–25.1) | 15.2 (12.9–17.8) | 19.3 (17.0–21.8) | 20.3 (17.1–24.0) |
| Diabetic (%) | 14.8 (14.0–15.6) | 14.1 (12.9–15.5) | 13.6 (11.6–15.9) | 15.9 (14.0–18.0) | 14.0 (11.9–16.3) | 17.6 (15.8–19.6) | 9.2 (7.3–11.6) |
| Hypertensive (%) | 22.1 (21.0–23.3) | 14.6 (13.1–16.3) | 20.2 (17.8–22.8) | 33.0 (30.6–35.6) | 23.2 (20.4–26.2) | 27.8 (25.3–30.6) | 16.9 (14.1–20.2) |
| Hypercholesterolemic (%) | 42.4 (41.2–43.6) | 40.6 (38.7–42.5) | 43.9 (40.7–47.1) | 47.6 (45.2–50.0) | 35.4 (32.1–38.7) | 44.2 (41.1–47.3) | 41.5 (37.1–45.9) |
| Cigarette use (%) | | | | | | | |
| Never | 61.6 (60.3–62.8) | 63.7 (61.6–65.7) | 70.4 (67.3–73.4) | 55.4 (52.4–58.2) | 76.6 (73.0–79.9) | 50.2 (47.2–53.2) | 64.6 (60.2–68.7) |
| Former | 17.4 (16.5–18.2) | 18.5 (17.0–20.1) | 14.9 (12.8–17.3) | 18.6 (16.9–20.5) | 11.4 (9.3–13.9) | 16.6 (14.5–18.9) | 22.1 (19.1–25.4) |
| Current | 21.1 (20.0–22.2) | 17.8 (16.1–19.7) | 14.7 (12.5–17.2) | 26.0 (23.6–28.6) | 12.0 (9.3–15.5) | 33.2 (30.5–36.0) | 13.3 (10.6–16.6) |

BMI, body mass index; CI, confidence interval; GED, general equivalency diploma; HEHS/SOL, Hispanic Community Health Study/Study of Latinos.
