# Supplementary material for: How Well Do U.S. Hispanics Adhere to the Dietary Guidelines for Americans? Results from the Hispanic Community Health Study/Study of Latinos
Source: Health Equity. 2019 Jul 11;3(1):319–27. doi: 10.1089/heq.2018.0105 (PMC6643200; doi:10.1089/heq.2018.0105)
Supplement: Supplemental data [file Suppl_TableS2.pdf]

Supplementary Table S2. Healthy Eating Index Total and Component Mean Scores for Hispanic/Latino Adults by Sex and Age, HCHS/SOL (2008–2011)[a]

		Sex		Age (years)		
HEI component	Range	Male ($n = 6229$)	Female ($n = 9404$)	18–30 ($n = 2647$)	31–50 ($n = 6600$)	51–74 ($n = 6386$)
HEI 1: Total fruit	0–5	2.43 (0.09)	3.15 (0.03)	2.17 (0.11)	2.68 (0.04)	3.09 (0.06)
HEI 2: Whole fruit	0–5	2.54 (0.06)	4.07 (0.44)	2.31 (0.05)	3.03 (0.20)	3.99 (0.09)
HEI 3: Total vegetables	0–5	3.10 (0.03)	3.43 (0.08)	2.89 (0.11)	3.22 (0.04)	3.40 (0.10)
HEI 4: Greens and beans	0–5	3.12 (0.09)	3.23 (0.23)	2.83 (0.13)	3.23 (0.14)	3.17 (0.10)
HEI 5: Whole grains	0–10	4.54 (0.01)	5.09 (0.04)	4.64 (0.18)	4.80 (0.10)	4.50 (0.06)
HEI 6: Dairy	0–10	5.25 (0.02)	6.33 (0.02)	5.66 (0.00)	5.54 (0.07)	5.86 (0.06)
HEI 7: Total protein foods	0–5	4.96 (0.01)	4.88 (0.03)	4.95 (0.00)	4.93 (0.01)	4.90 (0.02)
HEI 8: Seafood and plant proteins	0–5	3.89 (0.06)	4.06 (0.01)	3.12 (0.06)	4.00 (0.08)	4.27 (0.10)
HEI 9: Fatty acids	0–10	6.06 (0.17)	5.85 (0.11)	5.83 (0.12)	6.03 (0.13)	5.87 (0.00)
HEI 10: Refined grains	0–10	6.33 (0.03)	6.58 (0.13)	5.98 (0.28)	6.32 (0.27)	6.60 (0.02)
HEI 11: Sodium	0–10	3.39 (0.03)	4.17 (0.08)	3.62 (0.10)	3.75 (0.02)	3.65 (0.15)
HEI 12: Empty calories[b]	0–20	15.81 (0.11)	16.06 (0.08)	14.01 (0.10)	15.55 (0.23)	16.92 (0.12)
Total HEI score	0–100	61.44 (0.43)	66.90 (0.09)	58.01 (0.57)	63.07 (0.75)	66.23 (0.28)

[a]Adjusted by age and sex accordingly (mean age: 41.30, % male: 47.76). HEI components calculated based on multivariate NCI method (see statistical methods for details). Higher total score is indicative of a healthier diet.

[b]Calories from solid fats, alcohol (threshold >13 grams/1,000 kcals), and added sugars.

HEI, Healthy Eating Index; NCI, National Cancer Institute.
